# Neighborhood size and local geographic variation of health and social determinants

**DOI:** 10.1186/1476-072X-4-12

**Published:** 2005-06-01

**Authors:** Mohammad Ali, Jin-Kyung Park, Vu Dinh Thiem, Do Gia Canh, Michael Emch, John D Clemens

**Affiliations:** 1 International Vaccine Institute, SNU Research Park, San 4–8 Bongcheon-7 dong, Kwanak-gu, Seoul, Korea; 2 National Institute of Health and Epidemiology, No. 1 Yersin Street, Hanoi, Vietnam; 3 Robert Wood Johnson Foundation Health & Society Scholar, Columbia University, USA

## Abstract

**Background:**

Spatial filtering using a geographic information system (GIS) is often used to smooth health and ecological data. Smoothing disease data can help us understand local (neighborhood) geographic variation and ecological risk of diseases. Analyses that use small neighborhood sizes yield individualistic patterns and large sizes reveal the global structure of data where local variation is obscured. Therefore, choosing an optimal neighborhood size is important for understanding ecological associations with diseases. This paper uses Hartley's test of homogeneity of variance (F_max_) as a methodological solution for selecting optimal neighborhood sizes. The data from a study area in Vietnam are used to test the suitability of this method.

**Results:**

The Hartley's F_max _test was applied to spatial variables for two enteric diseases and two socioeconomic determinants. Various neighbourhood sizes were tested by using a two step process to implement the F_max_test. First the variance of each neighborhood was compared to the highest neighborhood variance (upper, F_max1_) and then they were compared with the lowest neighborhood variance (lower, F_max2_). A significant value of F_max1 _indicates that the neighborhood does not reveal the global structure of data, and in contrast, a significant value in F_max2 _implies that the neighborhood data are not individualistic. The neighborhoods that are between the lower and the upper limits are the optimal neighbourhood sizes.

**Conclusion:**

The results of tests provide different neighbourhood sizes for different variables suggesting that optimal neighbourhood size is data dependent. In ecology, it is well known that observation scales may influence ecological inference. Therefore, selecting optimal neigborhood size is essential for understanding disease ecologies. The optimal neighbourhood selection method that is tested in this paper can be useful in health and ecological studies.

## Introduction

Spatial filtering can be used to create smoothed maps of health and ecological patterns [1-4]. Since population distributions are highly heterogeneous in space, an ordinary point plot of all cases is not useful. Smoothing data by adjusting for the population at risk is necessary to identify areas with higher disease rates [5]. Smoothing disease data can provide the true relative risk of a disease across a study area [6]. There are other reasons to filter health and ecological data. Field survey data gathering systems usually generate errors. Filtering removes random noise caused by inaccurate records or mislocated cases [1,7,8]. There are many intervening factors at the individual level that may influence spatial processes of disease phenomena. For instance, an individual's biological or socioeconomic status may influence their health status. Neighbors usually have similar risk, particularly for environmentally related diseases, unless the spatial process of the disease is exclusively affected by individual-level characteristics. Also, some risk factors of diseases genuinely operate at the population level [9].

People do not live in isolation; they live in groups (neighborhood) that may influence their life style, health, and health seeking behavior. Thus, a neighborhood level study is sometimes essential to identify important public health problems and to generate hypotheses about their potential causes [9]. Twigg et al. showed that the behavioral practices of an individual are influenced by neighbors [10]. Some variables do not make sense at the individual scale and should be modeled as ecological variables. For instance, a household with a good sanitation system can be exposed to bad sanitation from neighbors. Ecological factors are more meaningful if the data are measured by neighborhood. Spatial filtering can be used to model such neighborhood level phenomena.

Ecological variables can be measured at different geographic scales from local to global. In ecology, it is well known that observation scales influence ecological inference [11-13]. Determining the neighborhood size (or the area) over which densities of the phenomena are estimated is important. A large neighborhood makes the data flat over the entire study area whereby important local level variation is obscured that could point to ecological associations. In contrast, a small neighborhood may reveal individualistic patterns [1], and that may not be useful for identifying ecological relationships with health outcomes. Defining an optimal neighborhood size is difficult [14,15]. Bailey and Gatrell [16] suggested exploring different sizes and looking at the variation at those scales to come up with an optimal neighborhood size. However, literature that describes methodologies for selecting the optimal size of neighborhood is scarce. Thus, one often chooses the scale arbitrarily, and the use of an arbitrary scale may yield spurious outcomes. This paper introduces a methodological approach for selecting the optimal neighborhood size that can be used to measure ecological variables and to investigate ecological links with local variation of diseases.

## Methodology

### The Study area

**Figure 1:**
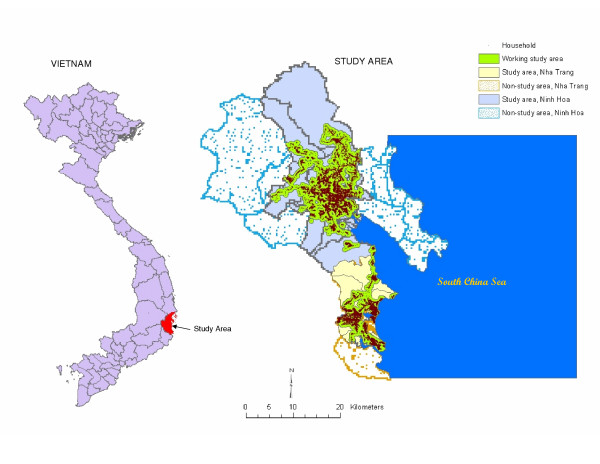
**The geographic features of the study area in Vietnam**. Map showing the geographic characteristics of the study area along with the geographic positing of the study area in Vietnam

Health and socioeconomic data of a study area in Khanh Hoa Province, on the coast of central Vietnam, were used to test the proposed method. The size of the study area is 740 square kilometers consisting of 33 communes in two districts: Nha Trang (151 square kilometres) and Ninh Hoa (589 square kilometres). A dynamic population database is maintained for the study area which is updated on yearly basis. In 2002, the population of the study area was 329,596, of which 54% of the population is from Nha Trang. We created a household-level geographic information system (GIS) database that includes a point for each active household (described below) in 2002. The household settlements are clustered, leaving a large portion non-inhabited land within the administrative boundaries, which led us to define household-based working study area by creating 500 meters radius buffer around each household point and dissolving boundaries between buffers (Figure 1). This resulted in a 394 square kilometres working study area for the entire population and 79 square kilometres for Nha Trang specifically.

### The GIS data

In 2003, we conducted a global positioning system (GPS) survey using handheld receivers to identify the geographic locations of every household in the study area. A base map of commune boundaries and other geographic features (e.g., rivers, roads, railways, lakes) was first acquired in digital format. The household GPS survey data were projected in the same geographic referencing system (i.e., Transverse Mercator) so that the household points could be accurately integrated with the study area base map. When several households shared a single structure or closely connected structures a single point was plotted. We received a list of 72,152 households from the census 2002. A total of 3,587 households could not be included in the GIS for a variety of reasons (e.g., migration out, living on a military base with no access, and household confirmation was not possible due to the absence of household members). Thus, the GIS was created for the 68,565 households, which were referenced spatially by 32,542 points. Several checks were made for missing households, misallocations, and wrong identification numbers, and the erroneous data were corrected. Finally, the household data were mapped in groups (the smallest geographic entity), and their positions were verified by ground-truthing.

### The attribute data

We used two social variables, religion and literacy (i.e., years of schooling), and two enteric disease incidence variables (i.e., shigellosis and *Vibrio parahaemolyticus*). The disease incidence data were obtained from a population-based passive surveillance system that was begun in January 1997 [17]. The socioeconomic data were obtained from a 2002 population survey. The survey data shows approximately 85% of the population is secular, 10% Buddhist, and 5% Christian. Only 11% had not attended school, 37% had received a primary education, and the rest (52%) completed secondary or higher education.

The *Vibrio parahaemolyticus *data were derived from the disease surveillance of the study area from 1997 to 1999. *V. parahaemolyticus*, a gram-negative, halophilic bacterium, inhabits marine and estuarine environments. The microorganism was first identified as a cause of food borne illness in Japan in 1950 [18]. A polymerase chain reaction (PCR) based method to detect the *toxR *sequence specific to *Vibro parahaemolyticus *was used to identify cases as reported elsewhere [19]. The shigellosis study was carried out for three years (2001–2003) in Nha Trang in collaboration with the Diseases of the Most Impoverished Program [20]. All shigellosis cases were detected through microbiological test of stool samples.

### Data categorization and manipulation

We categorized the two variables, literacy and religion, to define the social status of the study population. A person having six years of schooling or above was considered to be literate, and the religion was classified by Buddhist and non-Buddhist. Since the data were obtained at the individual level, the data were aggregated by spatially referenced household points. Neighborhood level data were then obtained for each of the spatially referenced points of household (32,542 points for the 329,596 individuals in 68,565 households) by aggregating household level data of surrounding neighbors using circular windows of various sizes. The neighborhood level social variables were estimated by the percentage of people living within neighborhoods, and the disease incidence variables were expressed in rates per 10,000 people. Our aim was to create a local-level neighborhood variable for these phenomena. Therefore, based on the working size of the study area and the spatial distribution of the population we set the minimum size to a 100-meter radius neighborhood and increased the size stepwise by 100 meters until a 2000 meter size was reached. This resulted in 20 different neighborhood sizes from which to select an optimal neighborhood size.

### Statistical analysis

Since the data from smaller neighborhoods are individualistic in nature, a high variance value is expected. In contrast, a low variance value is expected for larger neighborhoods. A high variance value means that data are local and low variance means that they are global. To select an optimal size of neighborhood that can ensure that the ecological data are neither local nor global, we used Hartley's test of homogeneity of variance [21] that evaluates variation in variances across neighborhoods. The Hartley's test statistic, *F*_*MAX*_, is calculated by



where

 = maximum value of the variances among groups

 = minimum value of the variances among groups

Under the null hypothesis, the test assumes that the variances are equal. The critical value (CV) was calculated under the F-distribution with (k, ***n***_*MAX *_- 1) degrees of freedom at α = 0.05. Here, k is the number of groups and ***n***_*MAX *_is the maximum sample size among groups.

The F_max _test involved two steps. First the variance of each neighborhood was compared to the highest neighborhood variance (upper, F_max1_) and then they were compared with the lowest neighborhood variance (lower, F_max2_). A significant value (means the value does not fall within the CV) of F_max1 _indicates that the neighborhood does not have a global structure of data, and in contrast, a significant value in F_max2 _implies that the neighborhood data are not individualistic. The neighborhoods that are between the lower and the upper limits are the optimal neighbourhood sizes.

## Results

There were 131 cases of *Vibro parahaemolyticus *in 127 household points in the entire study area for the three years of study (1997–1999), and 308 cases of shigellosis were observed in 295 household points for the year 2001 through 2003 in Nha Trang. Out of the total 329,596 population, 31,924 (9.7%) were Buddhists who were identified in 3,681 household points of the total study area. And, a total of 168,699 (51.2%) literate persons were observed in 30,069 household points.

**Figure 2:**
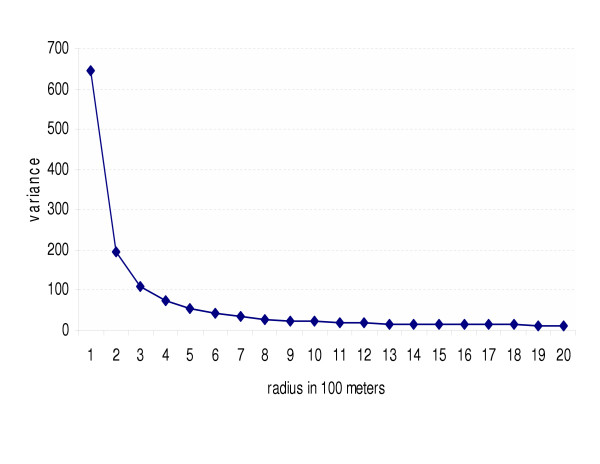
**The data variance by neighborhood size**. Graphical presentation of the data variances for the *Vibro parahaemolyticus *incidence in Khanh Hoa, Vietnam under various sizes of neighborhood.

The data variances for the *Vibro parahaemolyticus *incidence rates under various neighborhood sizes show a declining trend with an increase in neigborhood size (Figure 2). The rapid decline observed at smaller scales virtually disappears with larger neighborhood sizes. The pattern is similar for shigellosis as well as for both socioeconomic variables (figures not shown).

The test results for homogeneity variance of *Vibro parahaemolyticus *incidence rates under various neighborhood sizes are listed in Table 1. The F_max1 _test statistic at the level α = 0.05 shows a neighborhood size above 900 meters would reveal the global structure of the data, and the F_max2 _statistic shows that any neighborhoods below 200 meters would make the data too individualistic. Thus, the choice of optimal neighborhood lies between 200 and 900 meters. Considering the values of several parameters such as minimum population size, skewness and kurtosis of the incidence rate, we argue that a 500-meter neighborhood is optimal size for modeling the local variation of the disease incidence.

**Table 1:** Descriptive statistics and results of variance ratio (F_max_) test for the *Vibrio parahaemolyticus *incidence under various neighborhoods, Khanh Hoa Province, Vietnam, 1997–99. (n = 29,211)

r ^†^	Population size			Incidence Rate/10000 Population				Upper F_max _Test			Lower F_max _Test		
	
	Min	Max	Mean	Min	Max	Mean	Variance	F_max1_	DF_1 _*	CV_1 _**	F_max2_	DF_2 _*	CV_2 _**
100	1	1779	158	.00	1429.00	4.612	646.023	49.116	20	1.571	1.000	1	3.842
200	1	5143	492	.00	370.40	4.451	195.453	14.860	19	1.587	**3.305**	2	2.996
300	1	7372	914	.00	208.30	4.538	108.599	8.257	18	1.604	5.949	3	2.605
400	1	9252	1416	.00	161.30	4.533	73.509	5.589	17	1.623	8.788	4	2.372
**500**	3	12265	1971	.00	144.90	4.494	52.889	4.021	16	1.644	12.215	5	2.214
600	4	15784	2571	.00	227.30	4.486	42.481	3.230	15	1.667	15.207	6	2.099
700	4	19178	3236	.00	92.59	4.441	33.666	2.560	14	1.692	19.189	7	2.010
800	4	21949	3953	.00	52.91	4.434	28.671	2.180	13	1.720	22.532	8	1.939
900	4	24982	4711	.00	38.46	4.446	25.298	**1.923**	12	1.753	25.537	9	1.880
1000	4	26772	5508	.00	35.71	4.463	22.733	1.728	11	1.789	28.418	10	1.831
1100	6	28821	6324	.00	36.50	4.4939	20.647	1.570	10	1.831	31.289	11	1.789
1200	25	31691	7160	.00	36.50	4.5101	18.831	1.432	9	1.880	34.306	12	1.753
1300	50	34877	8029	.00	46.51	4.5099	17.453	1.327	8	1.939	37.015	13	1.720
1400	63	36311	8921	.00	37.17	4.5184	16.444	1.250	7	2.010	39.286	14	1.692
1500	63	37334	9832	.00	31.65	4.5266	15.655	1.190	6	2.099	41.266	15	1.667
1600	63	38471	10760	.00	28.33	4.5429	15.116	1.149	5	2.214	42.738	16	1.644
1700	63	39259	11693	.00	28.17	4.5510	14.593	1.109	4	2.372	44.269	17	1.623
1800	63	40278	12651	.00	27.70	4.5727	14.114	1.073	3	2.605	45.772	18	1.604
1900	63	41457	13657	.00	26.32	4.5990	13.639	1.037	2	2.996	47.366	19	1.587
2000	63	42492	14703	.00	26.32	4.6152	13.153	1.000	1	3.842	49.116	20	1.571

^† ^r = size of neighborhood in meter radius

* DF = degrees of freedom

**CV_1 _and CV_2 _= critical values at 95% confidence level for Upper F_max _and Lower F_max _respectively Bold figures in F_max1 _and F_max2 _are the upper and lower limit of optimal neighborhoods, and the bold figure in "r" column is the choice of optimal neighborhood size.

When looking at literacy, the F_max1 _test statistic shows a neighborhood above 600 meters would reveal the global pattern, and the F_max2 _test statistic demonstrates any neighborhoods below 700 meters would make the data individualist (Table 2). In this case, we believe that 700 meters is the optimal size because the difference between F_max1 _and CV_1 _is smaller than the difference between F_max2 _and CV_2 _of a 600-meter neighborhood. The summary statistics and test results of religious status under various neighborhood sizes are shown in Table 3. For religion, a 700-meter size neighborhood is also appropriate.

**Table 2:** Descriptive statistics and results of variance ratio (F_max_) test for the literacy status under various neighborhoods, Khanh Hoa Province, Vietnam, 2002. (n = 32,542)

r ^†^	Population size			Incidence Rate/10000 Population				Upper F_max _Test			Lower F_max _Test		
	
	Min	Max	Mean	Min	Max	Mean	Variance	F_max1_	DF_1 _*	CV_1 _**	F_max2_	DF_2 _*	CV_2 _**
100	1	1859	195	.00	100.00	50.226	221.986	3.262	20	1.571	1.000	1	3.842
200	1	5681	611	.00	100.00	50.191	167.826	2.466	19	1.587	1.323	2	2.996
300	1	8362	1143	.00	88.89	50.215	146.722	2.156	18	1.604	1.513	3	2.605
400	2	11276	1785	.00	83.33	50.229	134.220	1.972	17	1.623	1.654	4	2.372
500	2	15425	2504	.00	83.33	50.250	124.628	1.831	16	1.644	1.781	5	2.214
600	2	20047	3282	.00	80.50	50.241	117.075	**1.720**	15	1.667	1.896	6	2.099
**700**	2	23875	4146	.00	80.52	50.270	110.223	1.620	14	1.692	**2.014**	7	2.010
800	2	28601	5075	.00	80.46	50.296	104.018	1.528	13	1.720	2.134	8	1.939
900	2	33159	6055	.00	80.14	50.318	98.808	1.452	12	1.752	2.247	9	1.880
1000	4	36182	7081	15.70	75.96	50.333	94.737	1.392	11	1.789	2.343	10	1.831
1100	9	39576	8129	11.11	74.55	50.344	91.235	1.341	10	1.831	2.433	11	1.789
1200	25	43628	9194	15.97	73.14	50.355	88.216	1.296	9	1.880	2.516	12	1.752
1300	25	47769	10297	16.07	72.95	50.373	85.460	1.256	8	1.939	2.598	13	1.720
1400	25	49747	11427	16.52	72.44	50.386	82.739	1.216	7	2.010	2.683	14	1.692
1500	25	51108	12580	16.54	71.57	50.390	80.180	1.178	6	2.099	2.769	15	1.667
1600	66	52454	13750	16.54	70.89	50.394	77.521	1.139	5	2.214	2.864	16	1.644
1700	128	53725	14925	16.61	70.67	50.392	74.995	1.102	4	2.372	2.960	17	1.623
1800	138	55422	16133	16.61	69.93	50.393	72.569	1.066	3	2.605	3.059	18	1.604
1900	138	57913	17398	17.36	69.52	50.394	70.210	1.032	2	2.996	3.162	19	1.587
2000	148	59428	18708	17.75	68.81	50.412	68.054	1.000	1	3.842	3.262	20	1.571

^† ^r = size of neighborhood in meter radius

* DF = degrees of freedom

**CV_1 _and CV_2 _= critical values at 95% confidence level for Upper F_max _and Lower F_max _respectively Bold figures in F_max1 _and F_max2 _are the upper and lower limit of optimal neighborhoods, and the bold figure in "r" column is the choice of optimal neighborhood size.

**Table 3:** Descriptive statistics and results of variance ratio (F_max_) test for the ethnicity status under various neighborhoods, Khanh Hoa Province, Vietnam, 2002. (n = 32,542)

r ^†^	Population size			Incidence Rate/10000 Population				Upper F_max _Test			Lower F_max _Test		
	
	Min	Max	Mean	Min	Max	Mean	Variance	F_max1_	DF_1 _*	CV_1 _**	F_max2_	DF_2 _*	CV_2 _**
100	1	1859	195	.00	100.00	7.283	243.304	4.026	20	1.571	1.000	1	3.842
200	1	5681	611	.00	100.00	7.344	183.209	3.032	19	1.587	1.328	2	2.996
300	1	8362	1143	.00	100.00	7.383	153.024	2.532	18	1.604	1.590	3	2.605
400	2	11276	1785	.00	100.00	7.402	133.603	2.211	17	1.623	1.821	4	2.372
500	2	15425	2504	.00	100.00	7.467	121.327	2.008	16	1.644	2.005	5	2.214
600	2	20047	3282	.00	88.57	7.534	112.520	1.862	15	1.667	**2.162**	6	2.099
**700**	2	23875	4146	.00	88.57	7.609	105.353	**1.744**	14	1.692	2.309	7	2.010
800	2	28601	5075	.00	82.50	7.682	99.320	1.644	13	1.720	2.450	8	1.939
900	2	33159	6055	.00	75.00	7.719	93.729	1.551	12	1.752	2.596	9	1.880
1000	4	36182	7081	.00	69.49	7.745	88.828	1.470	11	1.789	2.739	10	1.831
1100	9	39576	8129	.00	63.73	7.761	84.867	1.404	10	1.831	2.867	11	1.789
1200	25	43628	9194	.00	62.40	7.776	81.459	1.348	9	1.880	2.987	12	1.752
1300	25	47769	10297	.00	62.40	7.793	78.140	1.293	8	1.939	3.114	13	1.720
1400	25	49747	11427	.00	61.91	7.801	74.610	1.235	7	2.010	3.261	14	1.692
1500	25	51108	12580	.00	60.84	7.820	71.456	1.183	6	2.099	3.405	15	1.667
1600	66	52454	13750	.00	58.06	7.851	69.027	1.142	5	2.214	3.525	16	1.644
1700	128	53725	14925	.00	50.55	7.873	66.823	1.106	4	2.372	3.641	17	1.623
1800	138	55422	16133	.00	46.79	7.891	64.531	1.068	3	2.605	3.770	18	1.604
1900	138	57913	17398	.00	44.54	7.907	62.339	1.032	2	2.996	3.903	19	1.587
2000	148	59428	18708	.00	39.71	7.915	60.426	1.000	1	3.842	4.026	20	1.571

^† ^r = size of neighborhood in meter radius

* DF = degrees of freedom

**CV_1 _and CV_2 _= critical values at 95% confidence level for Upper F_max _and Lower F_max _respectively Bold figures in F_max1 _and F_max2 _are the upper and lower limit of optimal neighborhoods, and the bold figure in "r" column is the choice of optimal neighborhood size.

The test results for the choice of optimal neighborhood size for shigellosis obtained from the Nha Trang subpopulation are shown in Table 4. The F_max1_test statistic reveals that a neighborhood size over 800 meters would produce a global pattern. On the other hand, the F_max2 _test statistic illustrates that a neighborhood below 300 meter would yield an individualistic pattern. Out of the choices between 400 and 800 meters, we suggest a 400 meter neighborhood size based on the criteria mentioned above for *Vibro parahaemolyticus*.

**Table 4:** Descriptive statistics and results of variance ratio (F_max_) test for shigella incidence under various neighborhoods, Nha Trang, Vietnam, 1999–2001. (n = 13565)

r ^†^	Population size			Incidence Rate/10000 Population				Upper F_max _Test			Lower F_max _Test		
	
	Min	Max	Mean	Min	Max	Mean	Variance	F_max1_	DF_1 _*	CV_1 _**	F_max2_	DF_2 _*	CV_2 _**
100	3	5692	1015	.00	333.30	6.041	197.436	25.397	20	1.571	1.000	1	3.842
200	3	17440	3223	.00	333.30	6.155	74.927	9.638	19	1.587	2.635	2	2.996
300	3	25689	6026	.00	57.14	6.112	38.808	4.992	18	1.605	**5.088**	3	2.606
**400**	10	34531	9388	.00	41.67	6.116	28.304	3.641	17	1.624	6.976	4	2.373
500	30	47539	13112	.00	57.47	6.102	22.808	2.934	16	1.644	8.656	5	2.215
600	33	61692	17105	.00	34.36	6.121	18.871	2.427	15	1.667	10.462	6	2.099
700	33	73490	21480	.00	35.46	6.129	16.566	2.131	14	1.692	11.918	7	2.010
800	87	88015	26126	.00	27.47	6.140	14.514	**1.867**	13	1.721	13.603	8	1.939
900	87	102106	30938	.00	22.87	6.148	13.374	1.720	12	1.753	14.763	9	1.881
1000	87	111244	35834	.00	31.15	6.158	12.651	1.627	11	1.789	15.606	10	1.831
1100	87	121667	40711	.00	19.67	6.137	11.774	1.515	10	1.831	16.769	11	1.789
1200	150	134222	45547	.00	18.28	6.116	11.057	1.422	9	1.881	17.856	12	1.753
1300	425	146883	50410	.00	17.64	6.119	10.536	1.355	8	1.939	18.739	13	1.721
1400	628	152901	55270	.00	16.40	6.135	10.079	1.297	7	2.010	19.589	14	1.692
1500	628	156963	60090	1.21	15.92	6.135	9.588	1.233	6	2.099	20.592	15	1.667
1600	628	161121	64829	1.77	15.92	6.123	9.104	1.171	5	2.215	21.687	16	1.644
1700	628	165020	69457	1.21	15.92	6.129	8.751	1.126	4	2.373	22.562	17	1.624
1800	628	170190	74078	2.73	15.92	6.135	8.411	1.082	3	2.606	23.474	18	1.605
1900	628	177965	78890	2.68	15.92	6.153	8.099	1.042	2	2.996	24.378	19	1.587
2000	628	182551	83821	2.98	15.92	6.171	7.774	1.000	1	3.842	25.397	20	1.571

^† ^r = size of neighborhood in meter radius

* DF = degrees of freedom

**CV_1 _and CV_2 _= critical values at 95% confidence level for Upper F_max _and Lower F_max _respectively Bold figures in F_max1 _and F_max2 _are the upper and lower limit of optimal neighborhoods, and the bold figure in "r" column is the choice of optimal neighborhood size.

**Figure 3:**
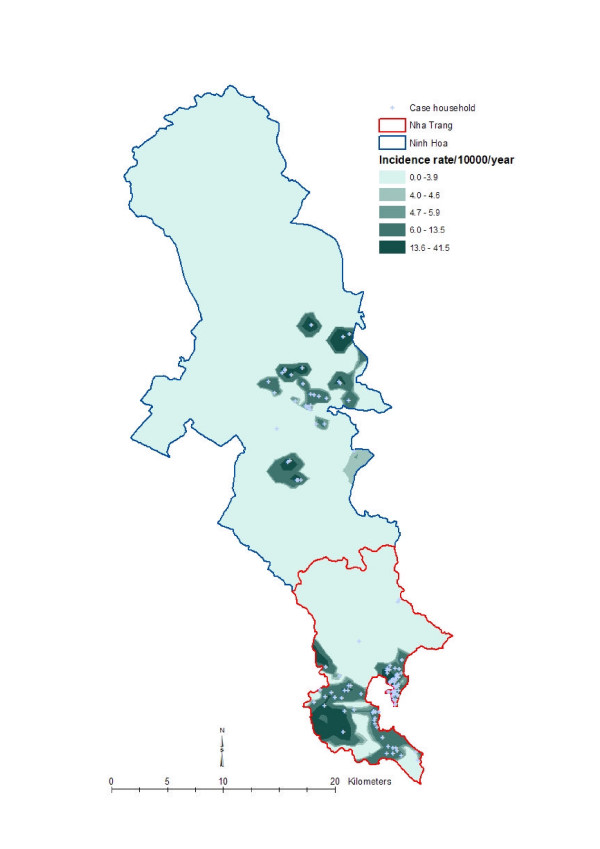
**Local geographic pattern of *Vibro parahaemolyticus *incidence rate in Khanh Hoa province, Vietnam**. The map was created based on the household point locations, thus the upper part of the study where no households are located have been omitted during the creation of the surface map. The lighter tones indicate lower *Vibro parahaemolyticus *incidence rate and the darker tones indicate higher *Vibro parahaemolyticus *incidence rate.

**Figure 4:**
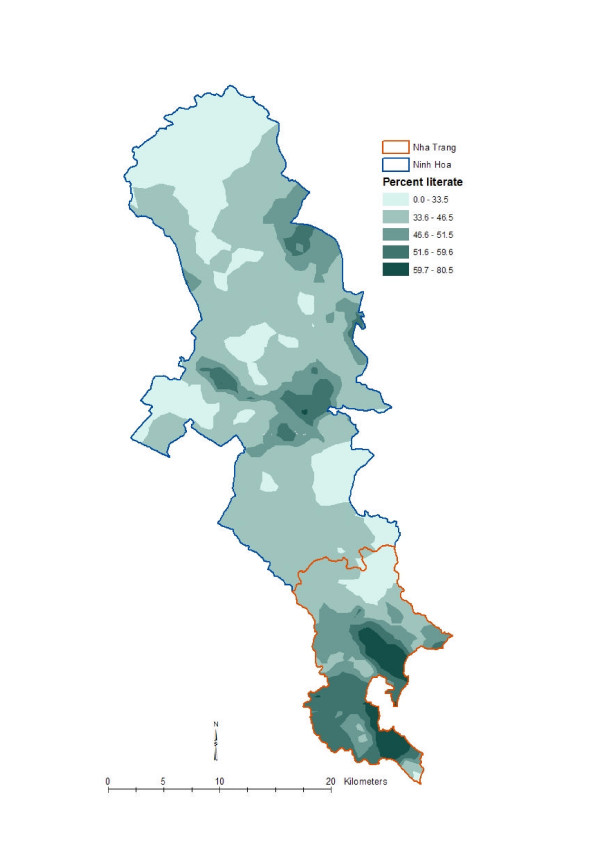
**Local geographic pattern of literacy status in Khanh Hoa province, Vietnam**. The map was created based on the household point locations, thus the upper part of the study where no households are located have been omitted during the creation of the surface map. The lighter tones indicate lower literacy status and the darker tones indicate higher literacy status.

**Figure 5:**
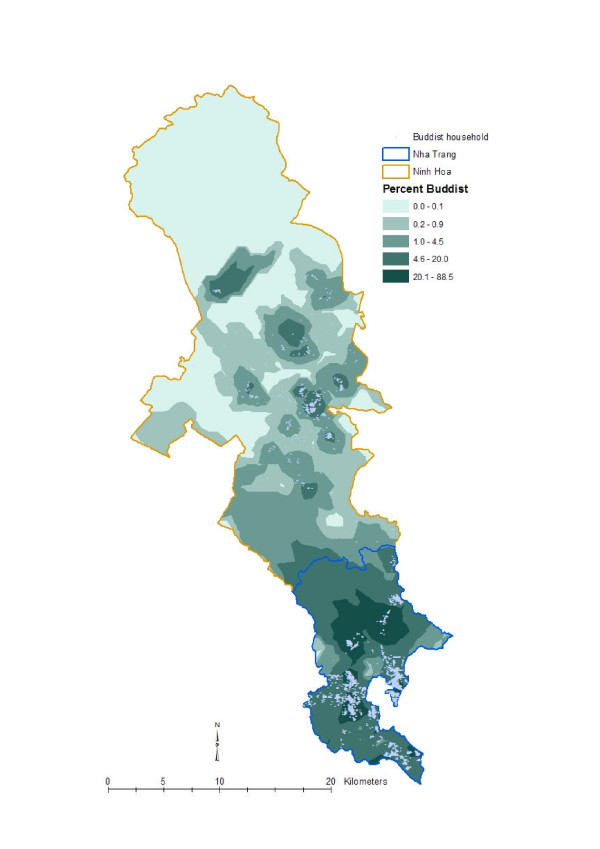
**Local geographic pattern of ethnicity status in Khanh Hoa province, Vietnam**. The map was created based on the household point locations, thus the upper part of the study where no households are located have been omitted during the creation of the surface map. The lighter tones indicate lower proportion of ethnically minority group and the darker tones indicate higher proportion of the ethnically minority group.

**Figure 6:**
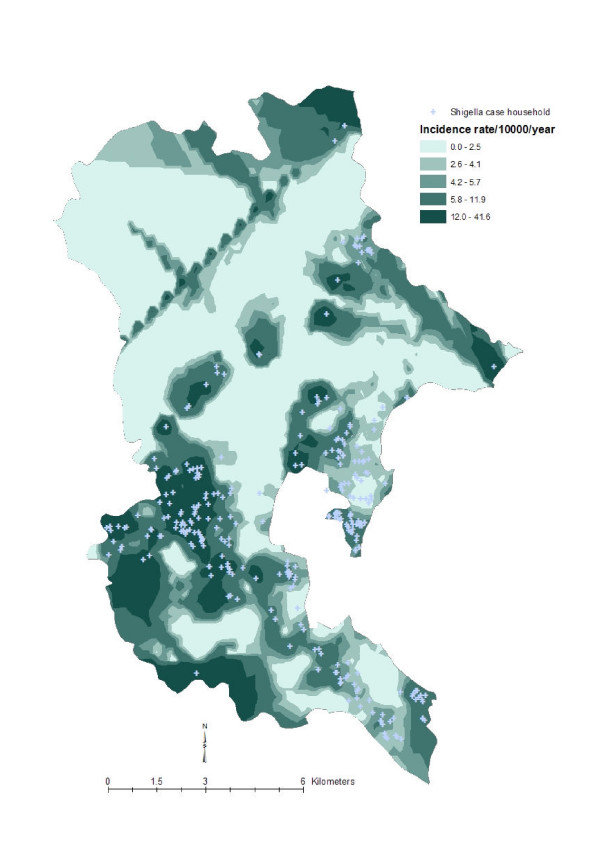
**Local geographic pattern of shigella incidence rate in Nha Trang, Vietnam**. The map was created based on the household point locations, thus the upper part of the study where no households are located have been omitted during the creation of the surface map. The lighter tones indicate lower shigella incidence rate and the darker tones indicate higher shigella incidence rate.

To get an understanding of local geographic variation of the disease and social variables, we created isopleth maps with the spatially smoothed data by using the optimal neighborhood sizes. Spatially smoothed data are more appropriate for disease and ecological mapping than the raw data [22]. A widely used geostatistical interpolation method called kriging [23,24] was used to create those maps. The maps ware produced as a quintile distribution for the respective phenomenon. Figure 3 shows the local geographic pattern of the *Vibro parahaemolyticus *incidence rate in Khanh Hoa province, Figure 4 shows the geographic pattern of literacy status in Khanh Hoa province, Figure 5 shows the pattern of religion in Khanh Hoa province, and Figure 6 shows the pattern of shigellosis incidence in Nha Trang. All of the maps show clear local geographic variation of the phenomena.

## Discussion and conclusion

The Hartley's F_max _test of homogeneity provides a solution for determining the optimal neighborhood size for modeling the local variation of health and social determinants. The methodological approach illustrates that the choice of optimal neighborhood is data dependent. *Vibrio parahaemolyticus *incidence requires a scale from 200 and 900 meters, and we argued that a 500 meter neighborhood is most appropriate based on the values of other parameters. The choice of neighborhood size for social variables (i.e., literacy and religion) ranged from 600 to 700 meters, and we suggested 700 meters for both. Similarly, out of the options between 300 and 800 meters for shigellosis incidence in Nha Trang, we suggest a 400 meter neighborhood. The maps produced using optimal neighborhood sizes show clear local geographic variation of the respective phenomenon suggesting the suitability of the approach. Since the ecological process may differ from one variable to another [25], different optimal neighborhood sizes are expected. The results of our analyses confirm this notion.

Measuring ecological data at a neighborhood scale to understand the spatial variability requires considerable knowledge of the phenomenon being measured [26]. For example, dissemination of an innovation may diffuse to close neighbors through literate persons. However, the media through which it diffuses is assumed spatially heterogeneous. For instance, a friendly neighborhood may accelerate the innovation, but disputes among neighbors may impede diffusion of the innovation. It would be ideal to assign weight for these social factors while measuring ecological variables, but that requires considerable knowledge about the spatial process of the phenomenon. For sanitation status, a poorly constructed latrine can be an important source of pollution by spreading fecal matter to nearby areas. Therefore, a distance decay weight can be applied here considering there is an inverse relationship from the source of pollution [27].

Since spatial filtering smoothes data, average errors may be inherent in the data [28]. Such ecological bias [29] can be more apparent in a predefined geographic area than within the natural boundary created through spatially smoothed data using optimal neighborhood modeling. Ecological bias may also be present when modeling variables with large neighborhood sizes.

One of the biggest problems in spatial epidemiology and ecological exposure assessment is in identifying geographic patterns [29] through spatial interpolation. Selection of an interpolation method has strong implications on the representation of spatial patterns as well as on the accuracy of interpolated data [30]. Interpolating the data based on spatially smoothed data obtained by an optimal neighborhood size could provide more accuracy in the local variation of the phenomena being measured. The optimal neighborhood may help ecological analysis in two ways: aggregating the data (both dependent and independent variables) using optimal neighbourhood scales and performing the analysis at the ecological level, or by limiting the dependent variable at the individual level, but attaching ecological covariates (obtained through optimal neighbourhood size) to each individual [31].

A scientifically validated method is required to assist geographic research [32], and to properly use GIS technology in health and ecological studies [33]. In our paper, we have outlined a method to choose optimal neighbourhood sizes for addressing local spatial variation of disease and social determinants. The method can be useful in health and ecological studies.
